# Effectiveness of interferon-beta and temozolomide combination therapy against temozolomide-refractory recurrent anaplastic astrocytoma

**DOI:** 10.1186/1477-7819-5-89

**Published:** 2007-08-04

**Authors:** Takamitsu Fujimaki, Hisato Ishii, Akira Matsuno, Hajime Arai, Tadayoshi Nakagomi

**Affiliations:** 1 Department of Neurosurgery, Teikyo University School of Medicine, Tokyo, Japan; 2 Department of Neurosurgery, Juntendo University School of Medicine, Tokyo, Japan

## Abstract

**Background:**

Malignant gliomas recur even after extensive surgery and chemo-radiotherapy. Although a relatively novel chemotherapeutic agent, temozolomide (TMZ), has demonstrated promising activity against recurrent glioma, the effects last only a few months and drug resistance develops thereafter in most cases. Induction of O^6^-methylguanine-DNA methyltransferase (MGMT) in tumors is considered to be responsible for resistance to TMZ. Interferon-beta has been reported to suppress MGMT in an experimental glioma model. Here we report a patient with TMZ-refractory anaplastic astrocytoma (AA) who was treated successfully with a combination of interferon-beta and TMZ.

**Case presentation:**

A patient with recurrent AA after radiation-chemotherapy and stereotactic radiotherapy was treated with TMZ. After 6 cycles, the tumor became refractory to TMZ, and the patient was treated with interferon-beta at 3 × 10^6 ^international units/body, followed by 5 consecutive days of 200 mg/m^2 ^TMZ in cycles of 28 days. After the second cycle the tumor decreased in size by 50% (PR). The tumor showed further shrinkage after 8 months and the patient's KPS improved from 70% to 100%. The immunohistochemical study of the initial tumor specimen confirmed positive MGMT protein expression.

**Conclusion:**

It is considered that interferon-beta pre-administration increased the TMZ sensitivity of the glioma, which had been refractory to TMZ monotherapy.

## Background

Treatment modalities for recurrent glioma are limited. Since surgery or local radiotherapy can be applied only to limited patients, a more systemic approach such as chemotherapy is used in most cases. Until recently, however, chemotherapy has had only a limited impact for control of recurrent glioma. A relatively novel chemotherapeutic agent, temozolomide (TMZ), has demonstrated promising activity against recurrent glioma in some patients, however the effects last only a few months and drug resistance develops thereafter in most cases[1,2]. Resistance to TMZ is considered to be mediated, at least to some extent, by a DNA repair enzyme, MGMT (O^6^-methylguanine-DNA methyltransferase), which is induced in the tumor [3]. Interferon-beta has been reported to suppress MGMT in an experimental glioma model [4,5]. Here we report the successful use of a combination of interferon-beta and TMZ for treatment of recurrent anaplastic astrocytoma after failure of TMZ monotherapy.

## Case presentation

**Figure 1:**
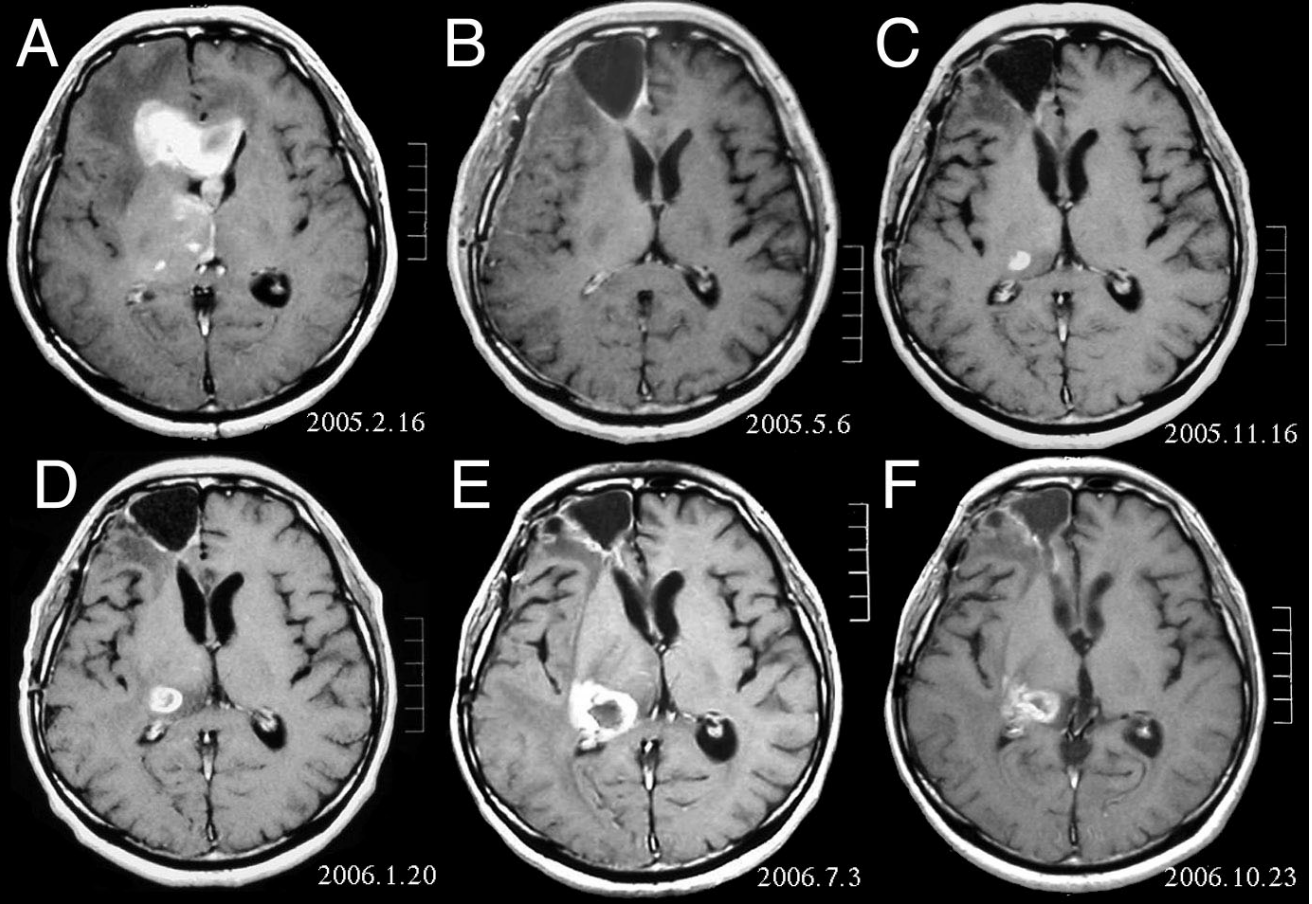
MRI of brain. (A) Initial MRI on February 16, 2005, shows a tumor in the right and left frontal lobe as well as the right thalamus. (B) MRI after surgery, radiation and chemotherapy. The tumor has completely disappeared except for slight enhancement adjacent to the surgical margin. (C) Recurrence of the thalamic tumor despite maintenance chemotherapy on November 16, 2005. (D) Increase in size of the thalamic tumor two months after stereotactic radiotherapy. (E) After 6 cycles of TMZ therapy, the thalamic lesion enlarged, and the patient developed dysarthria and hemiparesis. (F) After 2 courses of treatment with interferon-beta and TMZ, the tumor shows a partial response.

A 51-year-old man was found to have a diffusely infiltrative tumor in the bilateral frontal lobe and right thalamus (Figure 1A). The patient had undergone removal of a right frontal tumor, diagnosed as anaplastic astrocytoma (AA), on February 18, 2005. During local 60 Gy irradiation, chemotherapy consisting of procarbazine, nimustine hydrochloride (ACNU) and vincristine was given. After the radiation-chemotherapy, MRI showed complete disappearance of the lesion, including the thalamic tumor (Figure 1B). This combination chemotherapy was repeated every 3 months, but MRI on November 16, 2005, revealed recurrence in the right thalamus (Figure 1C). The patient received stereotaxic radiotherapy with 18 Gy (target volume 0.8 ml) for the recurrence in the thalamus, but follow-up MRI in January 2006 showed enlargement of the thalamic mass (Figure 1D). 

To differentiate radiation necrosis from recurrence, a fluorodeoxy glucose (FDG) PET study was performed. As the FDG-PET findings strongly suggested recurrence, TMZ chemotherapy was started. The patient was treated with the usual 5-day protocol repeated in cycles every 28 days, i.e. TMZ 150 mg/m^2 ^for the first 5 days, escalated to 200 mg/m^2 ^in the following cycles. Although the TMZ chemotherapy seemed to have some effect, the tumor continued to grow. After 6 cycles, the patient developed dysarthria and hemiparesis, and Karnofsky performance status (KPS) decreased from 100% to 70%. From the clinical course, the serial MRI findings (Figure 1C, 1D, 1E) and the previous FDG-PET data, we considered this was due to progression of the recurrent disease, and therefore we abandoned TMZ monotherapy. After application to the IRB, and with the informed consent of the patient, a combination of interferon-beta and TMZ treatment was started. On July 12, 2006, 3 × 10^6 ^international unit (IU)/body interferon-beta (Feron^®^) was given intravenously followed by 5 days of 200 mg/m^2 ^TMZ (Days 2 – 6). 

The patient's neurological symptoms improved after this first cycle, and MRI after the second cycle showed shrinkage of the tumor (Figure 1F). The patient's neurological symptoms also showed further concomitant improvement. This treatment was repeated every 28 days. After 8 cycles, the tumor showed further shrinkage, and since then the patient's condition has been improving, with a KPS of 100 in April 2007. During this treatment, no steroid has been administered and there have been no significant side effects exceeding grade 3 in terms of hematological and other clinical parameters.

### Immunohistochemical study to examine MGMT protein expression

The MGMT protein expression of the tumor specimen taken at the initial surgery was performed using immunohistochemical method as described previously [6]. Briefly, after antigen retrieval through autoclave treatment, the tissue sections were incubated in 0.3% hydrogen peroxidase for 30 min, then were incubated with the Ready-to Use anti-MGMT monoclonal antibody (clone MT3.1, Lab Vision, CA) overnight at 4°C. The sections were treated with a second biotinylated antibody (DAKO) then were incubated with streptavidin-biotin-peroxidase (SAB) complex (DAKO). Visualization was carried out with 3',3'-diaminobenzidine tetrahydrochloride (DAB).

**Figure 2:**
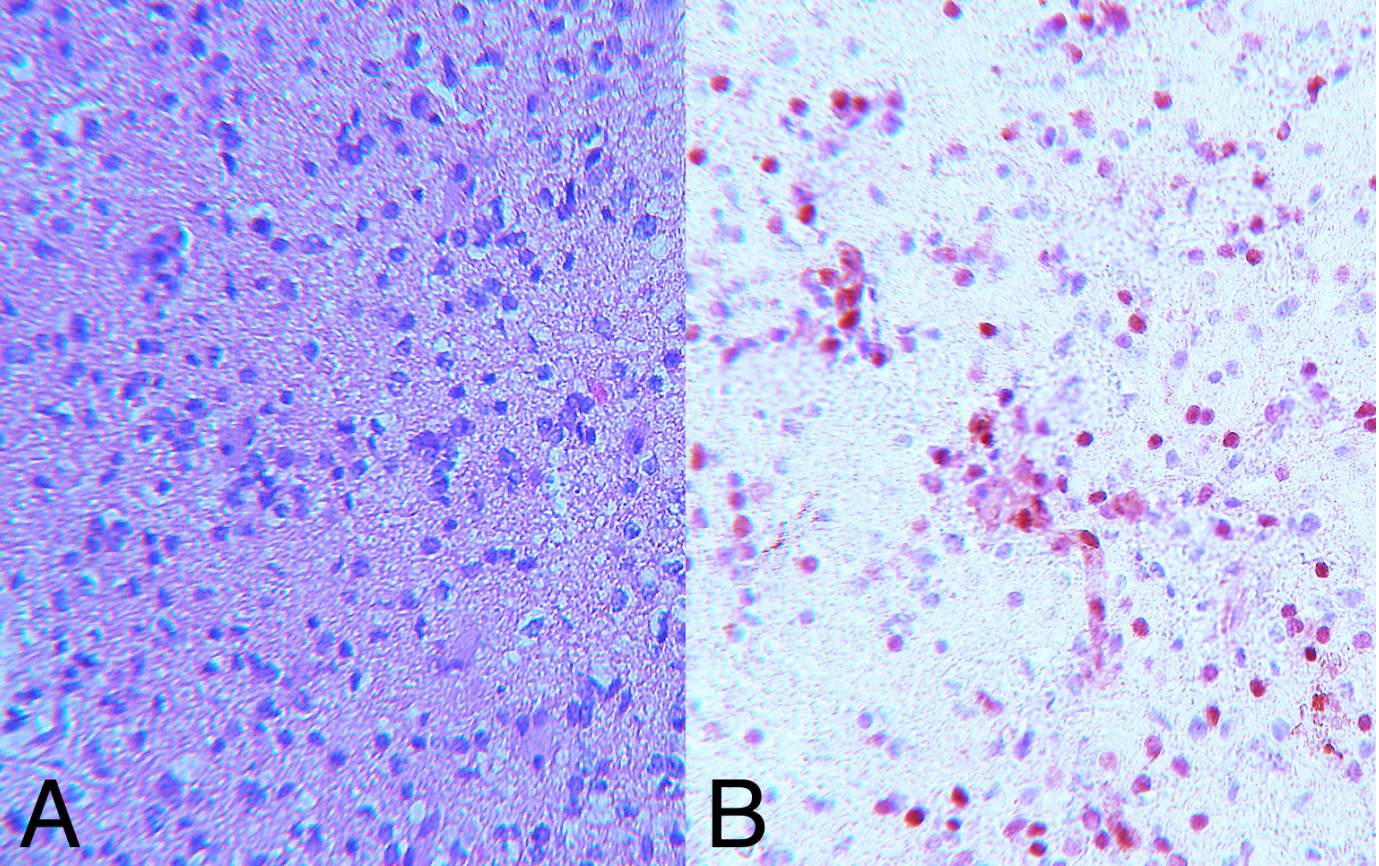
Photomicrograph. H&E staining (A) and immunohistochemical staining for MGMT protein (B) of the tumor resected at initial surgery. Forty-eight percent (± 3.7%) of the tumor cells express MGMT protein.

More than 40% of the tumor cells were positive for this staining thus it is confirmed that this tumor moderately[6]expressed MGMT protein (Figure 2).

## Discussion

We have reported a case of TMZ-refractory glioma that was treated successfully with interferon-beta and TMZ combination therapy. Although TMZ is one of the most effective chemotherapeutic agents for treatment of glioma recurrence, not all patients benefit from the drug. The response rate of glioblastoma (GBM) or AA to the standard TMZ protocol of 5 days in a 28-day cycle is 7–30% [1,2,7,8]. The median progression-free survival is only 2.1 months for GBM and 5.4 months for AA [2,7]. Once resistance has developed, there is no established management protocol. For TMZ, 21 consecutive days of treatment followed by 7 days of drug withdrawal ("21 days on and 7 days off") [9] or "7 days on and 7 days off" [10] or low-dose continuous treatment with TMZ have been tried [11]. Cyclophosphamide treatment has also been attempted [13]. However, all of these alternative treatments seem to have only limited effects. One of the mechanisms leading to TMZ resistance is the production of a DNA repair enzyme, MGMT [3,14]. High levels of MGMT activity in tumor cells create resistance by blunting the therapeutic effect of TMZ, leading to treatment failure. The tumor specimen of the present case taken at initial surgery showed that more than 40% of the cells expressed MTMT protein which might lead to the relative resistance to the TMZ monotherapy.

Interferon-beta has been used in Japan as an adjuvant therapy for some patients with malignant glioma [15]. It has a direct tumor-suppressive effect [16], and also acts as an immunomodulator [12] and anti-angiogenetic agent [17,18]. Recently, Natsume and Park showed that interferon-beta lowers the activity of MGMT and enhances the effect of TMZ *in vivo *and *in vitro *[4,5]. We administered interferon-beta before administration of TMZ to a patient whose recurrent AA had become refractory to standard TMZ monotherapy. The tumor showed a partial response after the second cycle, and thereafter the patient's condition improved and remained good for 8 months. Although interferon-beta itself has an antitumor effect against glioma, its effect is relatively mild, and one or two administrations of 3 × 10^8 ^IU/body is far less than that needed for adequate control. However, this dose may allow attainment of a serum concentration about one fourth of that shown to suppress MGMT activity in an *in vitro *setting [5]. It is speculated that although the tumor was initially resistant to TMZ monotherapy because of positive MGMT protein expression, the interferon-beta suppressed MGMT expression by the tumor, thus rendering it sensitive to interferon-TMZ combination treatment.

The MRI enhancement after stereotactic radiotherapy might have been due to a radiation effect. MRI is often useless for differentiating recurrence from radiation necrosis [19], and false positivity with FDG-PET has also been reported [20]. However, most previously reported tumors showing false positivity by FDG-PET or by MRI have been weakly enhanced or accompanied by marked and extensive central necrosis on MRI [19-21], unlike the present case. No previously reported tumor has been as extensive and with such a thick rim after stereotactic radiotherapy with a target volume as small as 0.8 ml. Moreover this thalamic lesion regressed after interferon-TMZ combination chemotherapy, without the use of steroid. Therefore we considered that this thalamic lesion represented genuine recurrence.

This is the first clinical report of effective treatment using a combination of interferon-beta and TMZ against TMZ-refractory glioma. This combination warrants further testing in a larger-scale clinical study.

## Conclusion

Combined treatment with interferon-beta and TMZ achieved a response in a case of TMZ-refractory recurrent AA. This approach may be a useful salvage therapy for patients with recurrent malignant glioma.

## Competing interests

The author(s) declare that they have no competing interests.

## Authors' contributions

TF: Involved in the diagnosis of the case, design of the treatment, submission of the protocol to the IRB, and drafting of the manuscript

HI: Involved in the treatment of the case

AM: Performed immunohistochemical study of the surgical specimen

HA: Involved in the surgery of the case

TN: Involved in the treatment of the case, and submission of the protocol to the IRB
